# Conservative treatment for anal incontinence

**DOI:** 10.1093/gastro/gou013

**Published:** 2014-03-18

**Authors:** Dan Carter

**Affiliations:** Department of Gastroenterology, Chaim Sheba Medical Center, Israel

**Keywords:** anal incontinence, loperamide, biofeedback

## Abstract

Anal incontinence (AI) in adults is a troublesome condition that negatively impacts upon quality of life and results in significant embarrassment and social isolation. The conservative management of AI is the first step and targets symptomatic relief. The reported significant improvement with conservative treatments for AI is close to 25% and involves prescribed changes in lifestyle habits, a reduced intake of foods that may cause or aggravate diarrhea or rectal urgency, and the use of specific anti-diarrheal agents. The use of a mechanical barrier in the form of an anal plug and the outcomes and principles of pelvic kinesitherapies and biofeedback options are outlined. This review discusses a gastroenterologist's approach towards conservative therapy in patients referred with anal incontinence.

## INTRODUCTION

Anal incontinence (AI), the involuntary loss of solid or liquid feces, is a common healthcare problem with a reported prevalence of between 1.6% and 15% [1–2]. AI has a major negative impact on quality of life and upon the activities of daily living [3], and is often accompanied by severe social restriction [4]. Within the individual's history, direct questioning is required concerning the complaint, with details relating to previous anorectal and colorectal surgery. Despite the fact that specific questionnaires about the severity of AI and its impact on quality of life are currently in worldwide use for many specialized conditions (neurological disease and spinal cord injury or *cauda equina* syndrome in particular), there are presently no standardized, comprehensive sets of questions designed to elucidate all the factors involved in AI. For the purposes of definition (and agreement) we can define several types of AI.

Passive AI has been defined as involuntary soiling or leakage of feces without patient awareness. This may be separable in some cases from fecal seepage. Flatus incontinence is the inability by the patient to control flatus. Both passive and flatus incontinence are primarily caused by internal anal sphincter dysfunction and, as such, are increasingly common complaints resulting from internal anal sphincterotomy for chronic anal fissure, an increased use of sphincter preserving techniques in cancer (with colo-anal anastomosis or intersphincteric rectal cancer resection) and as a result of novel hemorrhoid procedures that do not separate the anal cushions from the internal anal sphincter (e.g. PPH haemorrhoidopexy, ligasure hemorrhoidectomy and the like). Urge incontinence is the inability by the patient to defer defecation once an urge is perceived.

The history should asses:

- systemic and neurologic disorders
- stool consistency
- the presence of anorectal disease
- History of straining
- Detailed obstetric history including number of vaginal deliveries, use of assisted vaginal delivery, birth weight or dystocia, the use of episiotomy
- Prior anorectal surgery
- prior pelvic or anal irradiation
- associated urinary symptoms including urinary incontinence
- Associated pelvic organ prolapse
- Prior genito-urinary surgery

The aim of medical management is to treat the underlying disorders that cause diarrhea or constipation, to relieve troublesome and embarrassing symptoms, to restore bowel control and to improve the quality of life. When, however, these disorders cannot be readily identified or reversed, medical management is largely directed towards providing symptom relief. Various therapeutic modalities have been developed for the treatment of this condition (Table 1). The reported significant improvement attributed to these conservative treatments approaches 25% overall [2], such that conservative measures should be tried before consideration of any advanced surgical approach. These measures include changes in lifestyle habits, a reduced intake of foods that may cause or aggravate diarrhea or rectal urgency, urge suppression techniques and anti-diarrheal agents. This review outlines possible conservative therapies for use in AI, and their reported outcomes.

**Table 1.** Conservative modalities for the treatment of anal incontinence

Lifestyle habit modifications Cessation of smoking Weight loss Dietary strategies Sodium and protein reduction Caffeine restriction Dietary timing manipulation Elimination of aggravating foods (spicy foodstuffs, cabbage, onions) Selective lactose restriction Fiber supplementation insolubles (whole grain breads, cereals, nuts, beans, fruits and vegetables with skin and sweet corn), psyllium Adequate fluid intake Exercise régime Avoidance of drugs which exacerbate diarrhea Mechanical barriers- the anal plug Medications Anti-diarrheal treatments Phenylephrine gel Physical therapies Pelvic floor muscle training Biofeedback Electrical stimulation

## LIFESTYLE MANAGEMENT

Various potentially modifiable risk factors for AI have been reported. In this respect, current smoking was found to have a significantly deleterious effect on continence that appears to be unrelated to chronic obstructive pulmonary disease [5, 6]. Potential mechanisms of action include a direct influence on colonic transit (increasing total transit time but speeding rectosigmoid transit) and upon rectal compliance. In various epidemiological studies, obesity has also been reported to increase the overall risk of AI [7, 8]. This may be related to an increase in intra-abdominal pressure as well as to associated pelvic organ prolapse. In this respect, dietary intervention has not shown a significant difference in the fecal incontinence severity index (FISI) in these cases, compared with age- and sex-matched controls [9], with the effects of bariatric surgery showing AI improvement in selective cases presenting with morbid obesity [10, 11]. These results are, however, dependent upon the type of surgical procedure performed, with an actual worsening of AI following gastric bypass procedures, as compared with banding surgeries, which are independent of the amount of weight lost after surgery [12].

The effects of diet and dietary modification on AI have been examined in a few studies. A self-care change of diet or food avoidance, designed to manage AI, was reportedly successful in 67% of an elderly mixed cohort presenting with AI [13], and in more than one-third of AI patients in the general population [14]. Dietary strategies included modifying food types and eating patterns, with restriction of foods that contained caffeine or those substances perceived as producing flatus before leaving home. Amongst patients with AI, the consumption of lower levels of sodium and protein, with the institution of more dietary fiber, was found to be more common than in age-matched controls [15, 16], where systematic review has shown in observational studies and qualitative research protocols that self-care practices are effective, particularly in the elderly complaining of AI as a predominant symptom. These practices involve manipulation of dietary habits where skipping meals in an effort to influence incontinence—with dietary restriction of putative agents that worsened incontinence (e.g. fried and spicy foods, caffeinated foodstuffs, foods that increased flatus (e.g. cabbage, onions)—can influence AI-related quality of life. This may be supplemented by lactose avoidance in specific cases. Further, as AI occurs in association with fecal impaction or constipation, recommendations for an adequate intake of fluid to prevent hard stool consistency and constipation are also advised—although not tested.

Regarding fiber, a recent study of weight loss for incontinent and overweight women, showed that those with AI were 2.5 times as likely have a low fiber intake [17], with less clinical diarrhea when they ingested fiber as psyllium [18]. However, the data on the use of fiber conflicts with many studies showing an exacerbation of incontinence in some patients and this may well be the construct of the dietary fiber, where patients may benefit more from moderating their intake of foods containing largely insoluble fiber (whole grain breads, cereals, nuts, beans, fruits and vegetables with skin and sweet corn) [19], with a differential benefit in diarrhea-associated but not constipation-associated AI, particularly in the elderly population. Overall, the consumption of low amounts of fiber has previously been specifically reported in AI patients [20, 21], with improved outcomes in several studies [22–24]. Although fiber is used for constipation, it can also alleviate mild chronic diarrhea by absorbing water and increasing stool bulk, and possibly by creating the perception of decreased stool fluidity. The question of gastrointestinal symptom aggravation in AI patients receiving fiber supplements has been addressed in only one study, where there was a greater reported feeling of fullness in the fiber group as the only symptom that differed from symptoms reported by the placebo group during treatment [24].

## MECHANICAL TREATMENT: THE ANAL PLUG

**Figure 1.**
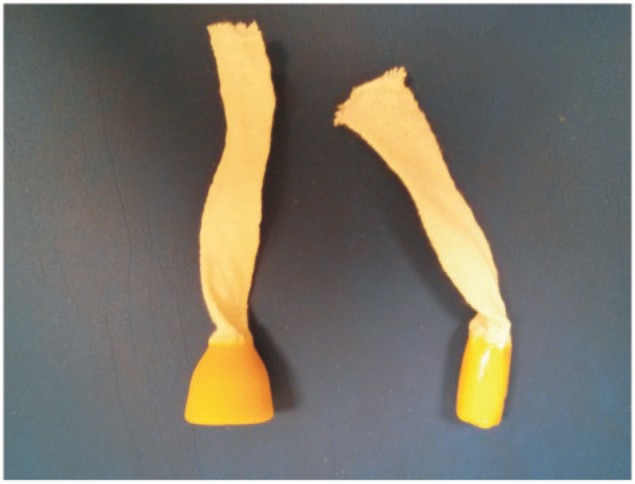
The Coloplast anal plug.

If incontinence persists despite treatment, a physical anal barrier to facilitate fecal containment may be a useful alternative. The use of pads for AI causes significant problems that are related to the difficulties in controlling the malodorous anal leakage, as well as associated skin problems. These problems can be overcome by the use of an anal plug, which is a special intra-anal device developed for containing fecal material in cases of AI (Figure 1). Historically, anal plugs were predominantly used in patients with neurological disorders; nowadays, however, the anal plug can be used for AI caused by other etiologies. The Conveen® anal plug (Coloplast, The Netherlands) is a single-use, disposable polyethylene plug with a compressed conical apex and a removal cord which, after insertion, settles into the anal canal for simple closure; it retails at a cost of about €4 at the time of writing.

Two studies have compared the use of anal plugs with standard treatment [25, 26]. In both studies, patients were allowed to choose between smaller or larger Coloplast plugs. Anal continence was achieved in 37% of the plug group and in none of the control group, although there was relatively poor compliance concerning plug use. In general, patients achieving anal continence reported greater satisfaction with treatment when using a plug than when not. In another similar study, the polyurethane anal plug (Conveen®: Coloplast) was found to be more useful than a polyvinyl-alcohol plug (Med. SSE-System, Germany) [27]. A recent systematic review found that, although the available data regarding any beneficial outcomes of the use of anal plug is limited, plugs may be helpful in alleviating the problems caused by incontinence, provided that they are tolerated and that patients persist with their use [28]. Overall, the data on plugs is subject to relatively poor methodology, limited follow-up and an eclectic group of patients in which the drop-out rates are predictably high. It is, however, a simple, inexpensive treatment associated with a reasonable patient satisfaction rate, that is probably under-utilized by clinicians, particularly in patients with AI and neurological disorders (spinal cord injury and some cases of spina bifida) who present with daily solid incontinence.

## MEDICAL THERAPY

Bowel disturbances—especially chronic diarrhea and irritable bowel syndrome—were found to be major risk factors for the development of AI [29]. On the other hand, AI can be the end result of fecal impaction and fecal overflow. Therefore, the rationale behind the pharmacological management of AI is to improve stool consistency, enhance anal sphincter function and promote complete rectal evacuation. Medical treatment targeting diarrhea and constipation has a major role in the conservative management of AI. Anti-diarrheal agents decrease intestinal motility and stool frequency, and modify stool consistency to a firmer controllable stool type. As a consequence, the number of AI episodes should decrease. Three different antidiarrheal medications have been tested for chronic diarrhea and AI; these are loperamide, codeine and diphenoxylate.

Loperamide is a synthetic opioid that does not cross the blood–brain barrier, and which has an excellent safety profile. Loperamide acts directly on the intestine to inhibit peristalsis, increasing small intestinal and mouth-to-cecum transit time and enhancing sphincter tone and resting pressure—thereby reducing urgency, stool volume and the frequency of bowel movements [30]. Loperamide may also reduce the sensitivity of the recto-anal inhibitory reflex and increases rectal perception in healthy subjects [31]. Codeine phosphate, another opiate derivative, functions similarly to loperamide, although it has a greater adverse effects profile in prolonged use, which is particularly marred by dependence. Diphenoxylate is another opioid derivative that inhibits intestinal motility and propulsion. Due to the possible side-effect of mild euphoria, atropine is usually added in sub-therapeutic doses to reduce the potential for overdose and abuse [32].

In an analysis of 13 studies that assessed the effects of antidiarrheal therapy on AI, anti-diarrheal drugs were found to be effective in terms of continence outcomes and measures of bowel function in relation to placebo [33]. Loperamide and codeine were associated with a higher percentage of solid stools and fewer adverse effects than diphenoxylate plus atropine [34, 35]. The greatest disadvantage of loperamide (which is the most commonly used drug) lies in the high potential for constipation and abdominal pain, particularly when the dose is increased rapidly. As a result, it should be started in a low dose (2–4 mg daily) and titrated according to symptoms.

As an alternative, topical phenylephrine gel has been used as a selective α1-adrenergic agonist that resembles naturally occurring catecholamines, and which can produce sympathomimetic effects. Phenylephrine can modulate the extrinsic innervation of the internal anal sphincter muscle, increasing anal sphincter tone and improving anal canal resting pressure. Four trials have tested topical phenylepinephrine gel [36–39], including patients with structurally intact anal sphincters and low resting anal pressures. Overall, patients were reported to be better when receiving the active drug, rather than the placebo, where more cases receiving phenylephrine gel achieved full continence or improved their incontinence symptoms. By contrast, in a recent study, clonidine (an a2-adrenergic agonist that can inhibit gastrointestinal motor activity by presynaptically inhibiting acetylcholine release from nerves in the myenteric plexus and at the neuromuscular junction) failed to alter bowel symptoms, fecal continence or anorectal functions in women with urge-predominant incontinence [40]. However, amongst patients with diarrhea, clonidine did increase stool consistency resulting in a borderline significant improvement in continence symptoms.

As fecal impaction can result in incontinence—with internal anal sphincter relaxation in response to pressure from the fecal bolus—the management in selected cases of impaction will influence reported AI. Impaired anorectal sensation, lower anal squeeze pressures, reduced integrity of the sphincters and/or pelvic floor musclature and neurogenic abnormalities are all factors that may promote incontinence in the presence of fecal impaction [41]. Constipation should be prevented by increases in fiber intake, fluid intake and physical activity. Constipation that does not respond to these measures may require the use of stool softeners, laxatives, tap-water enemas and rectal suppositories. In nursing home residents with AI related to fecal impaction, those who achieved rectal emptying had significantly lower rates of AI [42].

Patients with severe evacuation and post-defecatory incontinence may also benefit from daily rectal irrigation to help with colon evacuation. This approach may be supplemented by an antegrade irrigation system via an appendicostomy, ileostomy or caecostomy (which is discussed elsewhere in this Special Edition). Various types of equipment have been used for retrograde irrigation, including a stoma irrigation cone held in place manually against the anus [43], a mechanical pump [44], and specifically designed anal irrigation equipment [45, 46].

The useful effects of prebiotics (a general term describing a food ingredient that is not digested and which stimulates the growth and/or activity of colonic bacteria, including fructo- and galacto-oligosaccharides), probiotics (food supplements including *bifidobacteria* and *lactobacillus spp*., which contain live non-pathogenic and non-toxic microbes that have the potential to affect the colonic microflora) and synbiotics (products that combine prebiotics and probiotics) in chronic diarrhea are well documented [47]. However, hardly any research has been performed into the specific use of these agents in AI. In a group of patients who had undergone colonic surgery for colorectal cancer, the use of probiotics did not significantly change their daytime defecation frequency, night-time defecation frequency or their Wexner incontinence scores [48].

## PHYSICAL THERAPIES: SPHINCTER EXERCISES AND BIOFEEDBACK

Sphincter exercises, biofeedback therapy and electric stimulation are indicated for AI patients who do not respond to other conservative measures [49]. The striated muscles of the anal canal, the external anal sphincter and the puborectalis muscle, are amenable to formal pelvic floor muscle training (PFMT). These exercises include contractions of the pelvic floor musculature (the external anal sphincter and the puborectalis) whilst keeping the abdominal wall muscles relaxed, using a variable daily protocol up to 10–20 squeezes per day as a block, with 3–5 repeats a day. The purpose of the exercises is to enhance the strength, speed and endurance of voluntary anal sphincter contraction as well as to improve rectal emptying and contraction co-ordination.

**Figure 2.**
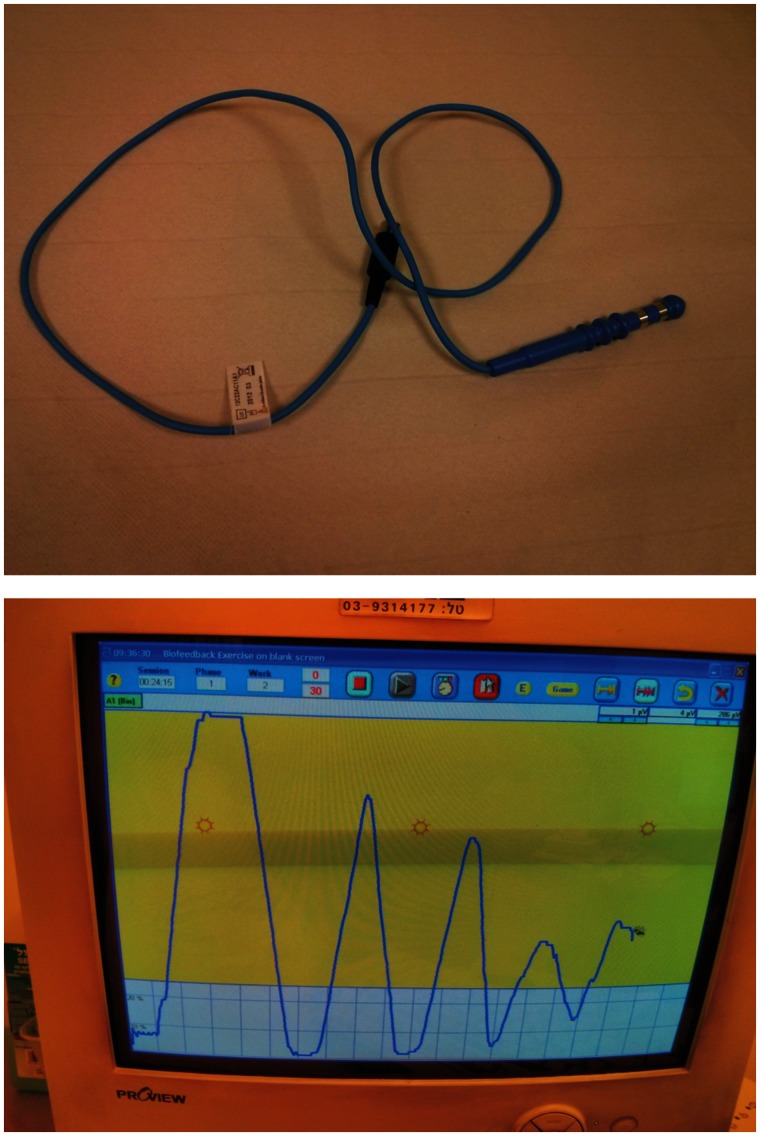
The top image shows an anal plug EMG electrode. The bottom image shows a contraction sequence trace (strong but non-sustained fatigable contractions) during biofeedback training when the patient is asked to hold stool and defer defecation.

Biofeedback therapy, which has been extensively reported in the surgical and nursing literature, is a therapeutic system based on instrumental learning, or operant conditioning. In this setting, a specific body function that may be only poorly perceived by the subject under normal conditions is measured by a technical device and demonstrated back to the subject. By the power of understanding, the subject may improve the specific exercised function (Figure 2). Early techniques concentrated on operant conditioning to enhance the voluntary contraction of the external anal sphincter, in response to the reflex inhibition of the internal anal sphincter when the rectum was filled [50]. Advanced techniques concentrated on teaching the subjects to discriminate smaller volumes of distension by use of a rectal balloon [51], and then to respond by contracting the external sphincter and abolishing delays between primary sensation and reaction. Other techniques focus on improving the strength or endurance of external anal sphincter contraction [52].

The therapeutic approaches (which are typically combined in treatment) include:

- **Strength training**: by the use of EMG skin electrodes, manometric pressure devices, intra-anal EMG, or anal ultrasound, the patient is encouraged to enhance squeeze strength and endurance.
- **Rectal sensitivity training**: in cases of rectal hyposensitivity, the subject is trained to feel the distension at progressively lower volumes, by repeated re-inflations of an intra-rectal balloon at progressively lower volumes, so that the subject can detect stool arriving at the rectum and has more time to find a toilet or use an anal squeeze function to delay unwanted defecation. In cases of rectal hypersensitivity, the same technique can also be used to teach the patient to tolerate progressively larger rectal distension volumes.
- **Co-ordination Training**: by use of a three-balloon system (one distension balloon in the rectum; the second and third smaller pressure-recording balloons are situated in the upper and lower anal canal), the subject observes the subsequent anal pressure drop due to the anorectal inhibitory reflux. The aim of this technique is to teach the patient how to counteract this drop by a voluntary anal squeeze that is sufficiently powerful and long enough to permit the resting pressure to return to its baseline level.

The literature, although extensive on biofeedback therapy in AI, is not particularly robust. More than 60 uncontrolled trials have been published on the use of biofeedback for the management of AI in adults, but only a few randomized studies have been reported. An early study by Norton *et al.* did not find any difference in the outcomes of various conservative interventions and those of exercises or biofeedback for AI [53]. Further studies, which recruited only subjects who failed to respond to conservative therapy, found a difference between exercise alone and exercise with the addition of rectal balloon [54], or EMG biofeedback [49, 55], and reported in favor of the addition of biofeedback therapy. Because of the small number of participants, however, there was not enough evidence to show a biofeedback advantage. The combination of biofeedback with PMFT does, however, appear superior to PMFT alone [56]. Further, the combination of electrical stimulation with biofeedback and exercises has been found to enhance the outcome of AI [51, 57]. Based upon the available data, no single method of biofeedback or exercises provides superior benefit over any other method, but biofeedback or electrical stimulation may offer an advantage over exercises alone if patients have previously failed to respond to other conservative management protocols.

Despite its relatively poor objective response, biofeedback rehabilitative treatment incorporating pelvic floor exercises is usually the first-line option for AI treatment that has not responded to simple dietary advice or medication, and virtually all patients referred to the specialist colorectal surgeon will have been through this form of therapy by the time of surgical referral [55]. The literature remains confused on these treatments because they are not standardized. There is further confusion regarding the description of various techniques, equipment variations, differences in outcome reporting, a lack of outcome predictors and variability in the length of patient follow-up. The likelihood of success is greater if there is sensory perception of rectal distension and if there is some effective external anal sphincter contraction ability. The patient who is motivated—with good cognition and an absence of significant depression—is also more likely to respond, particularly if there is a strong connection with the biofeedback technician [58].

Specific muscle training aimed at the levators (pelvic kinesitherapy) strengthens as well as co-ordinates the pelvic floor and is designed to supplement the stress abdominal-perineal reflex in those patients with pelvic floor descensus. Sensory training may also be used to support poor rectal discrimination and urgency with small volume rectal distension by retraining this sensory awareness and tolerance. The most impressive data utilize a directed multimodal rehabilitation program, where treatment is guided by anorectal pressure assessments [59], choosing biofeedback and pelviperineal kinesitherapy for those with low resting and squeeze activity, volumetric rehabilitation when there is a disordered rectal sensation or compliance and electrostimulation only if sensory improvement is desired. The role of this directed therapy for rehabilitation is unclear, but the results from the Italian group headed by Pucciani look more promising than regimented but unselected biofeedback protocols showing that nearly 90% of patients report a clinical improvement in their incontinence score and that up to 40% of cases become symptom-free.

## SUMMARY

In summary, the gastroenterologist is often the first specialist to see patients presenting with AI and will direct changes in lifestyle habits, fiber intake and specific anti-motility therapy in accordance with the presumptive underlying cause and severity of incontinence. Although outcome data on the physical therapies are conflicting, improved results might be expected from these treatments if they can be customized to cater for those cases with poor sphincter function or integrity or for disturbances in rectal compliance and sensitivity.

**Conflict of interest**: none declared.
